# CRISPR activation and interference as investigative tools in the cardiovascular system

**DOI:** 10.1016/j.biocel.2022.106348

**Published:** 2023-02

**Authors:** Melissa S. Carroll, Mauro Giacca

**Affiliations:** School of Cardiovascular and Metabolic Medicine & Sciences and British Heart Foundation Centre of Research Excellence, King’s College London, London UK

**Keywords:** CRISPR, DCas9, Transcriptional regulation, Cardiovascular

## Abstract

CRISPR activation and interference (CRISPRa/i) technology offers the unprecedented possibility of achieving regulated gene expression both in vitro and in vivo. The DNA pairing specificity of a nuclease dead Cas9 (dCas9) is exploited to precisely target a transcriptional activator or repressor in proximity to a gene promoter. This permits both the study of phenotypes arising from gene modulation for investigative purposes, and the development of potential therapeutics. As with virtually all other organ systems, the cardiovascular system can deeply benefit from a broader utilisation of CRISPRa/i. However, application of this technology is still in its infancy. Significant areas for improvement include the identification of novel and more effective transcriptional regulators that can be docked to dCas9, and the development of more efficient methods for their delivery and expression in vivo.

## 1 Introduction

The development of precise gene editing tools has become a fast-growing area of research, especially since the discovery of prokaryotic CRISPR RNA-guided Cas endonucleases (Jinek et al., 2012). These highly specific CRISPR/Cas systems are capable of targeting virtually any chosen DNA sequence in the mammalian genome, thus providing both a useful research tool and offering a potential therapeutic strategy for treating human diseases.

The cardiovascular system is one of the many areas that could benefit from gene editing. Mutations in specific genes cause over 40 cardiovascular disorders, the most prominent of which are hypertrophic and dilated cardiomyopathy (McKenna and Judge, 2021, Watkins et al., 2011), and inherited disorders of cardiac rhythm (Napolitano et al., 2018, Offerhaus et al., 2020). Several of the mutations underlying these conditions have a dominant inheritance and therefore selectively knocking out, correcting, or downregulating expression of the mutated gene presents an attractive therapeutic approach. This now appears attainable thanks to the specificity of CRISPR/Cas9 pairing to a complementary genomic DNA target. In addition, the possibility of selectively modulating expression of a given gene in adult animals by altering its transcription offers opportunities for novel phenotypic studies and provides an appealing alternative to current more laborious and time-consuming transgenic approaches.

Exploration of the cardiovascular system using CRISPR/Cas9 technologies has just started but appears to have a bright future ahead, provided that important technological issues are solved. Here we summarise the methods that are currently used to develop CRISPR/Cas9 tools, review the main applications of CRISPR activation and interference (CRISPRa/i) to modulate gene expression in the cardiovascular field to date, and highlight the current major limitations and future perspectives for this technology.

## 2 An overview of CRISPR/Cas9 technologies

Cas9 is the most commonly used CRISPR-associated protein containing two endonuclease domains (HNH and RuvC), which together can generate a double-stranded break (DSB) in DNA; HNH cleaves the DNA strand complementary to a guide RNA (gRNA) sequence, and RuvC cleaves the non-complementary strand (Jinek et al., 2012). Introducing a mutation in either of the endonuclease domains, typically a D10A mutation in RuvC or H840A mutation in HNH, creates a Cas9 nickase which is capable of making a single-stranded break (Trevino and Zhang, 2014). Mutations in both domains completely abolishes the endonuclease activity of Cas9, forming dead Cas9 (dCas9) (Larson et al., 2013, Qi et al., 2013).

Cas9 is often used in gene knockout experiments due to the frequent incorporation of insertion/deletion (indel) mutations following repair of Cas9-generated double-stranded breaks by non-homologous end joining (NHEJ) (Ran et al., 2013). However, precise gene editing can also be accomplished by providing an exogenous DNA template which can be incorporated into the target genomic locus by homology directed repair (HDR) (Ran et al., 2013). In contrast, dCas9 has been primarily used to alter transcription, enabling more sensitive changes in gene expression without creating potentially damaging double-stranded breaks. This is achieved by fusing dCas9 directly to one or more transcriptional effector domains or to a protein scaffold capable of recruiting multiple domains. Over the last few years, several laboratories have studied transcriptional regulation, epigenetic modifications, alterations to chromatin structure, and base editing by fusing dCas9 to a variety of effector domains.

## 3 dCas9-based transcriptional regulation

To target a specific genomic locus using Cas9 or dCas9, a gRNA is required, consisting of a CRISPR RNA (crRNA) complementary to approximately 20 nucleotides of the target DNA sequence, and a trans-activating RNA (tracrRNA), which forms a scaffold for Cas9 binding (Jinek et al., 2012, Qi et al., 2013). These two RNAs are commonly fused together to form a single guide RNA (sgRNA) (Cong et al., 2013). The DNA target sequence must include a downstream protospacer adjacent motif (PAM) sequence, which is recognised by the chosen Cas enzyme, allowing the sgRNA to base pair with the sequence located immediately upstream.

For CRISPRa/i technologies, the sgRNA is designed to bind the gene region immediately upstream or downstream of a transcriptional start site (TSS), bringing dCas9 and the fused transcriptional effector domains close to the promoter and thereby modulating gene expression (Larson et al., 2013, Qi et al., 2013). Previous research has identified ‘hotspot’ regions where the sgRNA can bind to stimulate the strongest transcriptional activation or repression. The exact region varies depending on the target gene and the CRISPRa/i system. Most studies report that targeting a region within − 400 to + 100 bp of the TSS is most efficient for both activation and interference (Gilbert et al., 2014, Konermann et al., 2015, Liao et al., 2017, Schoger et al., 2020). Within this region, Gilbert and colleagues found a peak in activation when targeting a region of − 400 to − 50 bp of the TSS using the dCas9-SunTag system in a high-throughput screening (Gilbert et al., 2014). The same researchers identified an optimal targeting region for repression using dCas9-KRAB which was − 50 to + 300 bp relative to the TSS, with a peak at approximately + 50 to + 100 bp, facilitated by physical blocking of the RNA polymerase by the CRISPRi machinery (Gilbert et al., 2014, Qi et al., 2013).

**Fig. 1:**
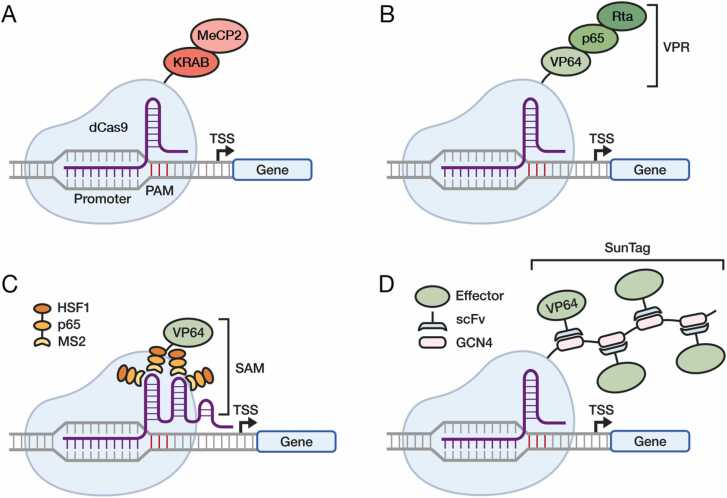
Main technologies for dCas9-based CRISPRa/i. A. dCas9-KRAB-MeCP2. B. dCas9-VPR. C. dCas9-SAM. D. dCas9-SunTag. The dCas9 protein is in pale blue, the CRISPR guide RNA is in purple. See text for description.

CRISPRa/i technologies date back approximately 10 years, with the first paper describing the use of dCas9 for transcriptional repression being published in 2013 (Qi et al., 2013). Since then, different variations have been developed to yield greater transcriptional changes. First generation CRISPRa systems included dCas9 fused to VP64, a tetramer of the herpesvirus factor VP16 joined by glycine-serine linkers (Maeder et al., 2013, Perez-Pinera et al., 2013), and one of the first CRISPRi systems was dCas9 fused to a Krüppel associated box (KRAB) domain (Gilbert et al., 2013). Effector domain screening has led to the identification of more efficient transcriptional effectors. For example, the fusion of MeCP2 to the KRAB domain, forming dCas9-KRAB-MeCP2 was identified as a strong transcriptional repressor (Yeo et al., 2018) (Fig. 1A). In addition, dCas9 is frequently fused to a tripartite activator consisting of VP64, p65 and Rta, referred to as VPR, which stimulates more robust transcriptional activation than VP64 alone (Chavez et al., 2015) (Fig. 1**B**).

Another common CRISPRa system takes advantage of dCas9 fused to VP64 and a Synergistic Activation Mediator (SAM) complex (Konermann et al., 2015). Unlike most CRISPRa/i systems that use a standard sgRNA, the dCas9-SAM system requires a modified sgRNA with two hairpin aptamers, that are capable of binding MS2 proteins. Each MS2 domain is fused to a p65 and HSF1 domain for strong activation. The SAM complex is formed following the recruitment of multiple MS2-p65-HSF1 to the modified sgRNA, and binding to dCas9-VP64 (Fig. 1C).

One of the most versatile CRISPRa/i technologies is the SunTag system, consisting of dCas9 fused to a flexible protein scaffold typically consisting of either 10 or 24 GCN4 peptides, to which single-chain variable fragment (scFv) antibodies can bind (Tanenbaum et al., 2014) (Fig. 1D). The scFv antibodies consist of the variable heavy and light chains of an immunoglobulin fused together by a linker to form a single polypeptide. The system was initially developed to produce stronger GFP signal by recruiting multiple copies of GFP to a target locus. However, this method has since been adapted by fusing the scFv to a variety of transcriptional activator, repressor, and epigenetic modification domains including VP64, DNA methyltransferase DNMT3A, and TET1, the last being involved in DNA demethylation (Huang et al., 2017, Morita et al., 2016, Tanenbaum et al., 2014).

A study by Chavez and colleagues aimed to fairly test the activation efficiency of different CRISPRa systems by targeting the same genomic loci with the same transfection conditions (Chavez et al., 2016). Initial trial experiments targeting two genes expressed at low or medium levels in HEK293T cells, *ASCL1* and *NEUROD1* respectively, identified dCas9-VPR, SAM and SunTag-VP64 systems as the strongest activators. Further tests in human cell lines identified that SAM was the strongest activator in HeLa, and SunTag was strongest in U2OS and MCF7, whereas similar activation was seen in mouse Neuro-2A and NIH-3T3 cell lines. Efficiency of transcriptional activation was improved by simultaneously targeting three sgRNAs to the promoter of *ASCL1*, *NEUROD1* and the highly expressed *CXCR4* gene.

Alternative CRISPRa/i systems have been developed by modifying the sgRNA length rather than mutating Cas9. Reducing the length of the sgRNA targeting sequence from 17–20 nt to 14–16 nt (called dead gRNAs) can guide nuclease active Cas9 (with or without fused transcriptional effectors) to a target region for transcriptional modulation while drastically reducing the introduction of DSBs (Dahlman et al., 2015, Kiani et al., 2015). Kiani and colleagues compared transcriptional activation of genes encoding three proteins (*ACTC1*, *TTN* and *HBG1*) in addition to the MIAT long-noncoding RNA (lncRNA) using Cas9-VPR and dCas9-VPR in combination with 14, 16 or 20 nt gRNAs. Cas9-VPR with a 14 nt gRNA increased expression of all targets to at least 40 % of the increase achieved by dCas9-VPR with a 20 nt gRNA (Kiani et al., 2015). Mutagenesis was observed when targeting the three protein encoding genes with 20 nt gRNAs, but the frequency of indels was reduced or abolished with 14 and 16 nt gRNAs (Kiani et al., 2015).

Another study by Dahlman and colleagues tested a range of sgRNAs with targeting sequences of 11–20 nt against the human haemoglobin 1 *(HBG1)* gene (Dahlman et al., 2015). In addition to shortening the sgRNA, two MS2 aptamers were incorporated into the sgRNA backbone, which can be bound by the MS2-p65-HSF1 (MPH) effector complex from the SAM system (Dahlman et al., 2015). Indel mutations were detected in cells transfected with active Cas9, the MPH complex and an sgRNA-MS2 with length 16–20 nt. Whereas 11–15 nt sgRNAs did not induce detectable indels but did increase mRNA expression of *HBG1* by 10,000-fold. The system was optimised for 14–15 nt sgRNA-MS2 for use with available Cas9 mouse models (Dahlman et al., 2015). This study also demonstrated the ability to achieve simultaneous gene activation and knockout, a key advantage of using dead sgRNAs in combination with nuclease active Cas9. A375 cells expressing active Cas9 and the MPH complex were transduced with a dead sgRNA-MS2 targeting *LPAR5*, a gene promoting drug resistance, and achieved over 600-fold increase in mRNA expression with maximum 0.85 % indel formation. These authors simultaneously targeted *MED12* and *TADA2B* tumour suppressor genes with a 20 nt sgRNA and achieved knockout in 33–36 % of cells with 67.4–91.5% indels, respectively (Dahlman et al., 2015). A disadvantage of this system is the lower gene modulation achieved when targeting certain genes compared to using 17–20 nt sgRNAs with dCas9, in addition to the introduction of indels (Kiani et al., 2015). To prevent off-target indel formation, 14-nt sgRNAs can be designed to competitively bind off-targets without introducing DSBs (Coelho et al., 2020).

Dead sgRNAs have been used in Cas9 mouse models in vivo. One study used the system optimised by Dahlman and colleagues consisting of 14–15 nt sgRNA-MS2 targeting a luciferase reporter, in combination with the MPH activation complex from the SAM system (Dahlman et al., 2015, Liao et al., 2017). This was packaged into AAV and co-injected with a luciferase reporter into the hind-limb of adult mice expressing nuclease-active Cas9, followed by electroporation into muscle cells. After 9 days, luciferase expression was detected in animals injected with 14–15 nt dead sgRNAs but not with 20 nt sgRNAs. In this study, the same constructs packaged into AAV9 were also administered to neonatal mice into the hind-limb muscles, directly into the brain and systematically via facial vein injection, and to adult mice via tail vein injection. Luciferase expression was detected when using a dead sgRNA against luciferase, with no expression in animals injected with a scrambled dead sgRNA control. The same system was also used to upregulate the endogenous follistatin (*FST)* gene, which resulted in increased muscle mass 12 weeks post injection (Liao et al., 2017).

## 4 Epigenetic modifications using CRISPRa/i

Epigenetic mechanisms such as DNA methylation and histone acetylation regulate many processes within a cell, affecting chromatin accessibility and gene expression. DNA methyltransferase (DNMT) inhibitors such as 5-aza-2’-deoxycytosine are often used to study the cellular effects of gene promoter demethylation (Christman, 2002, Oki and Issa, 2006). However, the non-specific nature of DNMT inhibitors can result in potential toxicity in vivo (Christman, 2002). DNMT3A is a DNA methyltransferase commonly combined with dCas9 to repress transcription by methylating cytosines at the promoter region of a target gene, forming 5-methylcytosine (5-mC) (Stepper et al., 2017). Combined with a sgRNA against a target region, DNMT3A initiates *de novo*, site specific methylation at this region; fusion to DNMT3L can also enhance the DNA methylation process (Chedin et al., 2002, Gowher et al., 2005). In contrast, a member of the Ten-Eleven Translocation dioxygenase enzyme family (TET1) is often used with dCas9 to increase transcription by catalysing the demethylation of DNA (Liu et al., 2016). Both domains can be fused directly to dCas9 by a flexible linker or fused to an scFv antibody for use with the SunTag system; the latter has been shown to produce more on-target methylation (Huang et al., 2017, Morita et al., 2016, Pflueger et al., 2018). Histone modifications can also be introduced in vitro to alter the chromatin state of DNA. For example, dCas9 fused to the histone demethylase LSD1 has been targeted to enhancer elements to repress gene expression (Kearns et al., 2015), and dCas9 fused to the transcriptional activator and histone acetyltransferase p300 is commonly directed to enhancer and promoter regions (Hilton et al., 2015).

Several of the above-mentioned systems have been used to target genes with clinically relevant pathogenicity when differentially expressed. For example, the promoter region of *BRCA1*, a tumour suppressor gene, was demethylated in vitro at specific regions using dCas9 fused to the catalytic domain of TET1 (TET1CD) for transcriptional activation (Choudhury et al., 2016). The use of TET1 was also tested in vivo in the brain of mouse foetuses by targeting a STAT3-binding site upstream of the gene encoding astrocyte-specific glial fibrillary acidic protein (GFAP) (Morita et al., 2016). This study demonstrates the efficiency of CRISPRa/i systems to introduce epigenetic modifications in vivo, paving the way for additional applications.

## 5 CRISPR a/i in cardiovascular research

There are currently limited studies that have applied CRISPRa/i to cardiovascular research. However, one in vivo proof-of-concept study took advantage of a mouse model expressing dCas9-VPR under the control of a cardiomyocyte-specific MYH6 promoter (Schoger et al., 2020). The aim was to target two genes in cardiomyocytes with pathogenic associations, *MEF2D* and *KLF15*. MEF2D is an isoform in the MEF2 family of transcription factors, which is highly expressed postnatally in the heart, along with MEF2A. These factors regulate the cardiomyocyte response to stress factors including pressure overload, with MEF2D-null mice being resistant to hypertrophy (Estrella et al., 2015, Kim et al., 2008). KLF15 is expressed at low levels in the neonatal heart and has been implicated as an inhibitor of cardiac hypertrophy and metabolic homoeostasis (Fisch et al., 2007, Leenders et al., 2012). The highest fold change in gene expression for both *MEF2D* and *KLF15* was achieved by simultaneously targeting a combination of four sgRNAs to non-overlapping regions of the promoter, approximately − 120 to − 200 bp upstream of the TSS, when tested in C3H/10T1/2 fibroblasts and C2C12 myoblasts. Synergistic activation by multiplexed targeting is a characteristic widely reported in these systems (Chavez et al., 2015, Chavez et al., 2016, Konermann et al., 2015). The expression of *KLF15* increased by over 10- and 50-fold in C3H/10T1/2 fibroblasts and C2C12 myoblasts, respectively, and by approximately 2-fold for *MEF2D* in both cell types, as validated by RT-qPCR. Following AAV9 delivery of sgRNAs to the heart, *MEF2D* overexpression led to a hypertrophic phenotype and *KLF15* transcription was increased but remained physiologically silenced.

The Schoger et al. study also identified other key features of CRISPRa for cardiovascular applications. AAV9 can efficiently deliver one or multiple sgRNAs to the heart and AAV dosage can be titrated to modulate gene expression, a benefit of CRISPRa over other Cas9 systems that deliver an uncontrolled copy number of exogenous transgenes to each cell. This study also found that controlling the concentration of dCas9-VPR, rather than that of the sgRNA, was more critical for long-term transcriptional activation, and off-target binding was rare, with only one event identified when targeting *MEF2D*. Finally, this study also highlighted a common observation for CRISPRa/i applications, namely that the efficiency of overexpression/downregulation is inversely proportional to the basal level of gene expression. More specifically here, *MEF2D* has high basal expression in the postnatal heart compared to *KLF15*, so the achieved fold change in transcriptional activation was lower. This observation suggests that there is a maximum limit to the transcriptional activation that can be achieved in each cell. The limiting steps that control this maximum threshold and whether this is cell type-specific remains to be understood.

Another study focused specifically on calmodulinopathy, an arrythmia syndrome mainly afflicting young individuals. A heterozygous missense mutation in any of the three genes encoding an identical 149 amino acid calmodulin protein (CALM1, CALM2 or CALM3), a ubiquitously expressed calcium ion sensor, can result in arrythmias including long QT syndrome (LQTS) (Kotta et al., 2018). Limpitikul and co-workers first created a representative model of the LQTS phenotype using induced pluripotent stem cell-derived cardiomyocytes (iPSC-CMs) with a D130G heterozygous missense mutation in CALM2 (Limpitikul et al., 2017). After validating this model, the authors used a CRISPRi system, dCas9-KRAB, to silence the wild-type and mutant version of CALM2, reversing the calmodulinopathy in the iPSC-CMs. This study provides preliminary evidence for the possible applications of CRISPRi in the development of cardiovascular disease therapeutics.

The use of CRISPRa/i systems in non-cardiac in vivo studies demonstrates the potential applications of this technology. For example, CRISPRa/i has been used in mouse models in vivo to target specific organs such as the brain (Zheng et al., 2018, Zhou et al., 2018) and in disease models such as hereditary tyrosinemia (Wangensteen et al., 2018), obesity (Matharu et al., 2019) and kidney fibrosis (Xu et al., 2018). Zheng et al. tested multiple CRISPRa systems and found that a combination of dCas9-SunTag with the effector domain components of the SAM system fused to scFv (scFv-p65-HSF1) was the most potent activator when targeting genes in the brain (Zhou et al., 2018). Other applications of the CRISPRa/i systems in vivo have been recently reviewed (Pandelakis et al., 2020, Schoger and Zelarayan, 2022).

## 6 Lineage reprogramming

Another useful application of CRISPRa/i is for lineage reprogramming experiments in vitro. Mouse embryonic fibroblasts were converted into induced neuronal cells by combining dCas9 with two VP64 domains and targeting endogenous genes *BRN2*, *ASCL1* and *MYT1L*, known as BAM factors (Black et al., 2016). In addition, the dCas9-SunTag system in combination with VP64 or p300 has been targeted to the promoter of endogenous *SOX2* and the promoter and enhancer of *OCT4* to reprogramme mouse embryonic fibroblasts into induced pluripotent stem cells (iPSCs) (Liu et al., 2018).

In another study, human foreskin fibroblasts (HFFs) were reprogrammed into induced cardiac progenitor cells (iCPCs) using the dCas9-SAM system. Increased transcription of endogenous *GATA4*, *HAND2*, *MEF2C* and *TBX5*, transcription factors that are essential to cardiomyocyte differentiation, was achieved using dCas9-SAM and sgRNAs against the promoter of each gene. The addition of upregulated MEIS1 facilitated cardiac reprogramming by inducing cell cycle arrest in G2/M. The iCPCs expressed cardiac-specific genes including *NKX2–5*, *cTNT* and α-actinin, but displayed a disorganised sarcomeric structure. These iCPCs showed the potential to differentiate in vitro into cardiomyocytes, smooth muscle cells and endothelial cells (Wang et al., 2020).

More recently, CRISPRa reprogrammed cells have been tested in vivo as a treatment for myocardial infarction (MI). One study used dCas9-SAM to reprogramme mouse tail-tip fibroblasts into CRISPR-induced cardiovascular progenitor cells (ciCPCs) by upregulating cardiac transcription factors, including *GATA4*, *NKX2–5* and *TBX5* (Jiang et al., 2022). Following MI in mice, ciCPCs were injected into the heart between the infarct and border zone. The cells differentiated into cardiomyocyte-like cells, leading to reduced adverse remodelling such as left ventricular dilation, reduced scar formation, and increased ejection fraction compared to a sham control. Another study used dCas9-VP64 to upregulate *GATA4*, *MEF2C*, *NKX2–5*, *HAND2* and *TNNT2* in rat cardiosphere-derived cells (CDCs) (Sano et al., 2022). Injection of the activated CDCs into the infarct border zone improved left ventricular ejection fraction and reduced scar formation, compared to non-activated CDCs and PBS controls. However, low cell retention and engraftment remains challenging for this therapeutic approach for MI.

## 7 CRISPRa/i challenges and future perspectives

CRISPRa/i technologies are versatile systems, capable of efficiently modulating the transcription of endogenous genes. As such, they provide a useful alternative to other gene regulation approaches, including gene knock-out by standard CRISPR/Cas9, gene overexpression by exogenous cDNA or mRNA transfection/transduction, or downregulation of endogenous mRNA by RNA interference.

A common method to overexpress a protein of interest is to deliver a gene expression construct encoding cDNA or an ORF, which is then transcribed and translated by the cell. Advantages of CRISPRa compared to exogenous overexpression is the upregulation of endogenous gene expression with natural post-translational processing, and the ability to upregulate large, difficult to clone genes (Kampmann, 2018). However, not all genes can be efficiently targeted by CRISPRa/i. For example, studies have observed that genes with medium or high basal expression experience less substantial transcriptional upregulation than genes expressed at low levels, potentially due to a cellular mechanism imposing an upper limit to gene upregulation to prevent damage (Chavez et al., 2015, Chavez et al., 2016, Konermann et al., 2015). Additionally, the expression of a specific gene isoform cannot be modulated if the isoforms are under the control of the same promoter, and unlike gene knock-in methods, genes of a different species cannot be expressed, (e.g., a human gene in an animal model).

A common method for downregulation of a target gene is instead RNA interference (RNAi). This is a homology-dependent mechanism in most eukaryotic cells involving the regulation of gene expression by short double-stranded RNAs. An advantage of CRISPRi compared to RNAi is silencing at the endogenous DNA level in the nucleus rather than at the mRNA level in the cytoplasm, enabling nuclear transcripts such as lncRNAs to be downregulated. There are many commercially available RNAi reagents available to target nearly any gene of interest, whereas CRISPRa/i databases are relatively new and are based on algorithms predicting the most efficient gRNAs for CRISPRa/i. However, many endogenous promoters are poorly annotated and there are ample differences in the optimal targeting region for each individual gene. As a consequence, the currently available databases are often used as a starting guide, and multiple gRNAs used singularly or simultaneously must be tested to verify effective up- or down-regulation of each gene of interest (Horlbeck et al., 2016).

There are still several issues with CRISPRa/i systems that remain challenging. First, CRISPRa/i is subject to the same concerns as standard CRISPR/Cas9 in terms of target specificity, as the sgRNA can bind to off-target sites that have sequence homology to the target sequence due to its ability to tolerate a few mismatches (Fu et al., 2016). In this respect, one benefit of dCas9 systems is that they do not introduce damaging indels, and the possibility of altering transcription is low unless the off target is near a gene promoter (Kampmann, 2018).

Second, there is evidence of immunity to Cas9 originating from certain bacterial species (Nelson et al., 2019). In one study, high instances of human infection with *Staphylococcus aureus (Sa)* and *Streptococcus pyogenes (Sp)* resulted in approximately 78% and 58% of human donors having antibodies against SaCas9 and SpCas9 respectively (Charlesworth et al., 2019). In addition, this study identified that 78% of donors had anti-*Sa*Cas9 T cells and 67% had anti-*Sp*Cas9 T cells. This highlights a potential challenge with using Cas9 or dCas9 in vivo, as the immune system may elicit an immune response that could clear the cells expressing these proteins.Table.1.

**Table 1:** Main studies that have used dCas9-based CRISPRa/i systems for gene activation or repression.

CRISPRa/i system	Target gene (s)	In vitro/In vivo	Outcome	Reference
dCas9-VP64	VEGFA, NTF3 (human)	In vitro (human HEK293)	Activation	(Maeder et al., 2013)
dCas9-VP64	IL1RN, NANOG, HBG1/2, MYOD1, VEGFA, TERT, IL1B, IL1R2 (human) ASCL1 (human and *Mus musculus*)	In vitro (human HEK293T, mouse primary embryonic fibroblasts)	Activation	(Perez-Pinera et al., 2013)
dCas9-VP64	SIM1, MC4R *(Mus musculus)*	In vitro (mouse Neuro-2A) and in vivo (mouse)	Activation	(Matharu et al., 2019)
dCas9-VPR	MIAT, NEUROD1, ASCL1, RHOXF2, TTN, ACTC1, NGN2 (human) ACTA1, ACTC1, TTN, TUNA *(Mus musculus)* MTK, CECA1 *(D. melanogaster)* GAL7, HED1 *(S. cerevisae)*	In vitro (human HEK293T, mouse Neuro-2A, *Drosophila* S2R+, yeast strain *W303*)	Activation	(Chavez et al., 2015)
dCas9-VPR	MEF2D, KLF15 *(Mus musculus)*	In vivo (mouse heart)	Activation	(Schoger et al., 2020)
dCas9-SAM	ASCL1, MYOD1, NEUROG2, VEGFA, HBG1, TERT, IL1B, IL1R2, MYC, ZFP42, LIN28A, SOX2, NANOG, KLF4, POU5F1, long intergenic non-coding RNAs (lincRNA) (human)	In vitro (human HEK293FT, A375)	Activation	(Konermann et al., 2015)
dCas9-SunTag-VP64	CXCR4, CDKN1B (human)	In vitro (human HEK293, U2OS, K562)	Activation	(Tanenbaum et al., 2014)
dCas9-SunTag-VP64	Reactivation of latent HIV-1 (human)	In vitro (human C11, J-Lat, ACH2, HEK293T, TZM-bl)	Activation	(Ji et al., 2016)
dCas9-SunTag-VP64	MYC, TNFRSF1A, SLC7A11, TP53 *(Mus musculus)*	In vivo (mouse liver)	Activation	(Wangensteen et al., 2018)
dCas9-SunTag-TET1CD	STAT3-binding site of GFAP, H19, RHOXF2B, CARD9, SH3BP2, CNKSR1 *(Mus musculus)*	In vitro (mouse embryonic stem cells, neuronal precursor cells, A549) and in vivo (mouse brain)	Activation	(Morita et al., 2016)
dCas9-SunTag-p65-HSF1	Exogenous mCherry, endogenous genes ASCL1, NEUROG2, NEUROD1, ACTA1, DKK1, SLC6A4, RNF43, BCL2, ZNRF3, PRDM16, HBB, GRM2, LNS2, IL10, SLC7A11 and lncRNAs MIAT, HALGR, FENDRR, LNCPINT *(Mus musculus)*	In vitro (human HEK293T, mouse Neuro-2A, primary astrocytes and embryonic fibroblasts) and in vivo (mouse brain)	Activation	(Zhou et al., 2018)
dCas9-VPR, SAM, SunTag-VP64	TTN, HBG1, MIAT, TUNAR, RHOXF2, ACTC1, ASCL1, NEUROD1, CXCR4 (human) HBB-BH1, TTN *(Mus musculus)* Wingless, Twist *(D. melanogaster)*	In vitro (human HEK293T, HeLa, U2OS and MCF7, mouse Neuro-2A and NIH-3T3, *Drosophila* S2R+)	Activation	(Chavez et al., 2016)
dCas9-VP64, dCas9-p300	IL1RN, MYOD, OCT4, mammalian beta-globin locus control region (HBE, HBG, HBD, HBB) (human)	In vitro (human HEK293T)	Activation	(Hilton et al., 2015)
dCas9-TET1CD	BRCA1 (human)	In vitro (human HeLa, MCF7)	Activation	(Choudhury et al., 2016)
High-fidelity dCas9-TET3CD	RASAL1, EYA1, LRFN2, KLOTHO (human)	In vitro (human kidney cells, TK173, TK188 fibroblasts, and HK2 epithelial cells, mouse primary kidney fibroblasts and renal tubular epithelial cells) and in vivo (mouse kidney)	Activation	(Xu et al., 2018)
dCas9-KRAB	CD71, CXCR4 (human)	In vitro (human HEK293, HeLa)	Repression	(Gilbert et al., 2013)
dCas9-KRAB	CALM1, 2, 3 (human)	In vitro (human iPSC-CMs)	Repression	(Limpitikul et al., 2017)
dCas9-KRAB	Including GAS5, H19, MALAT1, NEAT1, TERC, XIST (lncRNAs) (human)	In vitro (human K562)	Repression	(Gilbert et al., 2014)
dCas9-KRAB	SYT1, VAMP2, SNAP25, STX1A, STX1B, DOC2A, DOC2B *(Mus musculus)*	In vitro (mouse primary neurons) and in vivo (mouse brain)	Repression	(Zheng et al., 2018)
dCas9-KRAB, dCas9-KRAB-MeCP2	CANX, CXCR4, CHK1, SEL1L, ARPC2, MAPK3, BRCA1, SYVN1, BLM, GZMM, MAPK3, RHOA, CHEK1, CHEK2, ARPC2, TERC, XIST (human)	In vitro (human HEK293T, HAP1, SH-SY5Y)	Repression	(Yeo et al., 2018)
dCas9-LSD1, dCas9-KRAB	OCT4, TBX3 *(Mus musculus)*	In vitro (mouse embryonic stem cells)	Repression	(Kearns et al., 2015)
dCas9-SunTag-DNMT3A	HOX5A, BACH2, KLF4 (human)	In vitro (human HEK293T)	Repression	(Huang et al., 2017)
dCas9-DNMT3A, dCas9-SunTag-DNMT3A	UNC5C, CCDC85C intron, SHB intron, MIR152, GAD1 intron 3, NRF1 binding sites (human)	In vitro (human HEK293T, HeLa, MCF7)	Repression	(Pflueger et al., 2018)
dCas9-DNMT3A-DNMT3L (DNMT3A3L)	EpCAM, CXCR4, TFRC (human)	In vitro (human SKOV-3, HEK293)	Repression	(Stepper et al., 2017)
VP64-dCas9-VP64	BRN2, ASCL1,MYT1L *(Mus musculus)*	In vitro (mouse embryonic fibroblasts)	Reprogramming mouse embryonic fibroblasts into induced neuronal cells	(Black et al., 2016)
dCas9-TET1, dCas9-DNMT3A	BDNF, MYOD *(Mus musculus)*	In vitro (mouse C3H10T1/2, embryonic stem cells, fibroblasts) and in vivo (mouse brain, skin epidermis)	Reprogramming fibroblasts into myoblasts	(Liu et al., 2016)
dCas9-SunTag-VP64/p300	OCT4, SOX2, KLF4, c-MYC, NR5A2, GLIS1, CEBPA *(Mus musculus)*	In vitro (mouse tail tip fibroblasts, embryonic fibroblasts)	Reprogramming mouse embryonic fibroblasts into iPSCs	(Liu et al., 2018)
dCas9-SAM	GATA4, HAND2, MEF2C TBX5 (human)	In vitro (human foreskin fibroblasts)	Reprogramming human fibroblasts into induced cardiac progenitor cells (iCPCs)	(Wang et al., 2020)
dCas9-SAM	GATA4, NKX2–5, TBX5, HAND2, MESP1, BAF60C, ISL1, GATA6, SRF, HAND1/2, IRX4 *(Mus musculus)*	In vitro (mouse primary fibroblasts)	Reprogramming mouse tail-tip fibroblasts into CRISPR-induced cardiovascular progenitor cells (ciCPCs)	(Jiang et al., 2022)
dCas9-VP64	GATA4, NKX2–5, MEF2C, HAND2 and TNNT2 *(Rattus norvegicus)*	In vitro (primary neonatal rat hearts to generate cardiospheres) and in vivo (rat heart)	Cardio-specific differentiation factor activation in cardiosphere-derived cells (CDCs)	(Sano et al., 2022)

Third and foremost, the method of delivery remains a challenge. The gold standard for delivery of CRISPR components to organs such as the heart in vivo is by AAV vectors. This is due to their relatively low immunogenicity (Verdera et al., 2020) and ability to stay mainly episomal in non-dividing cells (Zacchigna et al., 2014). Cardiomyocytes are non-dividing cells and therefore the AAV remains expressed throughout the lifespan of the infected cells, which may be beneficial if a long-lasting effect is required. However, the < 5 kb capacity limit of AAV vectors creates a challenge to package all the CRISPR components into one vector, specifically the larger Cas9 and dCas9 components. The use of split AAV vectors, for example using the intein-mediated protein splicing system, might overcome this issue (Lim et al., 2020, Villiger et al., 2018, Zhi et al., 2022). For experimental purposes, there has been an increase in CRISPRa/i mouse models with the large dCas9 components incorporated into the genome, often with Cre-inducible expression (Gemberling et al., 2021, Wangensteen et al., 2018, Zhou et al., 2018). Tissue specific expression can be achieved by delivering CRISPRa/i components under the control of a tissue-specific promoter, and using AAV serotypes with tissue tropisms.

In conclusion, CRISPRa/i technology is a potentially powerful tool to study the effects of gene regulation, but the possibility of extensively utilising this technology in vivo, especially for therapeutic applications, is still in its infancy. Significant areas for improvement include the identification of novel and more effective transcriptional regulators that can be docked to dCas9, and the development of more efficient methods for their delivery and permanent expression in the target cells.

## Data Availability

No data was used for the research described in the article.
